# PPARs Activity Affects the Hatchability Through Lipid Metabolism Regulation in Silkworm, *Bombyx mori* L.

**DOI:** 10.3390/biom15040492

**Published:** 2025-03-27

**Authors:** Xia Xu, Chunguang Cui, Xin Du, Jine Chen, Xiuling He, Linbao Zhu, Chengjie Hu, Fang Xu, Chenkai Ma, Shaofang Yu, Xingjian He, Houhui Song, Yongqiang Wang

**Affiliations:** 1 College of Animal Science and Technology, College of Veterinary Medicine, Zhejiang A&F University, Hangzhou 311300, China; 2 Institute of Sericulture and Tea, Zhejiang Academy of Agricultural Sciences, Hangzhou 310021, China

**Keywords:** PPARs activity, lipid metabolism, hatchability, *Bombyx mori* L.

## Abstract

Lipid metabolism serves as the primary energy source for organisms. Silkworm eggs for spring use are divided into two types: autumn-produced eggs for next spring rearing (AS) and spring-produced eggs for next spring rearing (SS). Production practice revealed significant differences in hatching rates between these two types of silkworm production strain QiufengA. In this study, we identified differentially expressed genes (DEGs) primarily enriched in energy metabolism pathways. In particular, the PPARs are involved in energy regulation through lipid metabolism. Furthermore, both AS and SS contained the same eight long-chain fatty acids but in different amounts. Interference with PPARs activity in silkworm eggs disrupted the expression of key genes in this pathway, resulting in a significant decrease in hatching rate. Additionally, knockdown of the pathway key gene *BmPlin4* led to the reduction in lipid droplets. In conclusion, PPARs regulates the hatching rate of silkworms mainly by affecting lipid metabolism. This study proved the importance of PPARs for hatching and identifies them as potential target genes for population control.

## 1. Introduction

Silkworm (*Bombyx mori* L.) is a model lepidopteran insect and holds significant economic importance, having been domesticated for over 5000 years [1,2,3]. China leads global cocoon production, contributing approximately 80% of the world’s total output [4]. Most current silkworm production strains are bivoltine, with egg development entering a dormant phase during early embryogenesis, a state referred to as diapause [5]. Bivoltine silkworms are typically found in temperate regions where the climate allows for two complete life cycles within a single year. To resume development and enable hatching in alignment with production schedules, diapause must be terminated through physical methods (e.g., temperature control) or chemical treatments (e.g., acid immersion). The spring climate in China is exceptionally favorable for high-yield cocoon production, making it the optimal season for sericulture [6].

**Figure 1:**
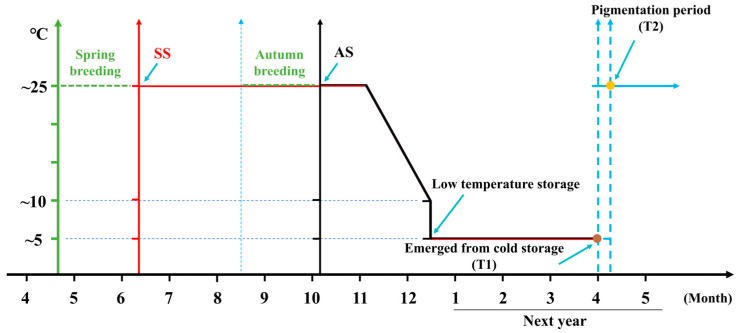
The developmental process of QiufengA. Green was the breeding time, red was the SS protection time, and black was the AS protection time. The brown circle represents removal from cold storage (T1); the orange circle was the body pigmentation stage (T2). AS was autumn-produced eggs for next spring rearing, SS was spring-produced eggs for next spring rearing.

Silkworm eggs designated for spring rearing are categorized into two types: autumn-produced eggs for next spring rearing (AS) and spring-produced eggs for next spring rearing (SS), both of which are preserved through double refrigeration method [7]. The AS was maintained at 25 °C starting from October, after which the temperature was reduced by 1 °C every two days. By December, the temperature had gradually decreased to the natural ambient temperature of 10 °C. Subsequently, the AS was transferred to 5 °C for storage to be used for rearing in the following spring. Similarly, the SS was kept at 25 °C from the time of egg laying until September. Starting from October, the SS underwent the same temperature treatment as the AS (Figure 1) [8]. The differing storage durations of AS and SS eggs lead to variations in hatching rates, particularly in the QiufengA strain, which is widely cultivated in China for its superior cocoon yield and quality. These differences are likely attributed to variations in energy metabolism during embryonic development, ultimately influencing hatching success.

Embryonic development requires a continuous supply of nutrients and energy expenditure to sustain growth and cellular activities [9]. During silkworm embryogenesis, both catabolic and anabolic processes are highly active, as cells proliferate and differentiate into various tissues, consuming substantial amounts of nutrients such as vitellogenin to support the developmental process [10,11]. Peroxisome proliferator-activated receptors (PPARs), a group of transcription factors within the nuclear receptor superfamily, regulate gene expression upon ligand activation [12]. PPARs influence embryonic development through energy regulation and cellular metabolism [13,14,15,16]. There are three PPARsubtypes: PPARα, PPARβ/δ, and PPARγ, which collectively maintain lipid homeostasis by regulating the transcription of target genes [17]. Specifically, PPARα is involved in fatty acid oxidation and energy metabolism, PPARβ/δ regulates glucose and lipid metabolism, and PPARγ promotes adipocyte differentiation [18]. These receptors are essential for maintaining lipid balance during cellular development and differentiation [11,12,13,14]. The regulation of lipid metabolism is fundamentally governed by the composition and concentration of fatty acids [19]. Fatty acids are classified into three primary categories: saturated fatty acids (SFA), monounsaturated fatty acids (MUFA), and polyunsaturated fatty acids (PUFA), each playing distinct roles in biological processes [20]. The specific types and relative abundance of these fatty acids significantly influence metabolic pathways and developmental processes [21]. Among SFAs, palmitic acid and stearic acid are the predominant forms [22]. Elevated levels of SFA have been associated with reduced messenger RNA abundance, protein content, and low-density lipoprotein receptor activity, ultimately affecting metabolic efficiency [23,24]. Oleic acid, as the most representative MUFA, demonstrates substantial beneficial effects on growth and development [25]. Linoleic acid, an essential PUFA, can exert inhibitory effects on normal development when present in excessive concentrations [26]. The oxidation of different fatty acids is crucial for the precise regulation of lipid metabolism. Consequently, comprehensive analysis of fatty acid profiles in silkworm eggs is vital for advancing our understanding of lipid metabolism dynamics and energy regulation mechanisms.

In silkworm production, the hatching rate is a pivotal determinant of cocoon yield and economic benefits. Although most silkworm strains exhibit comparable hatching rates between AS and SS, the QiufengA strain demonstrates a particularly notable disparity. In this study, we have revealed that the differences in the hatching rate of silkworms were attributed to the PPARs. These findings highlight the critical role of PPARs in egg hatching processes and provide valuable theoretical insights for enhancing sericulture production efficiency and economic outcomes.

## 2. Materials and Methods

### 2.1. Silkworm Strain and Cell Line

The silkworm production strain QiufengA is bred and provided by Zhejiang Academy of Agricultural Sciences, including autumn-produced eggs for next spring rearing (AS) and spring-produced eggs for next spring rearing (SS). Silkworm eggs were incubated at 25 °C and 80% RH for 10 days. The 1–3 instar larvae were reared on fresh mulberry leaves at 28 °C, and the 4–5 instar larvae at 25 °C and 75% RH [27]. To break diapause and ensure continuous rearing of QiufengA, the eggs were treated with hydrochloric acid to remove diapause. Then, 20 h after laying, the eggs were immersed in 15% hydrochloric acid at 46 °C for 5 min and then transferred to 25 °C until hatching. Additionally, the BmN cell line, derived from a *B. mori* ovary tissue, was utilized in this study [28]. The cells were cultured in TC-100 medium (LVN1013, Livning, Beijing, China) supplemented with 10% fetal bovine serum (FBS) in 25 cm^2^ Petri dishes at 27 °C.

### 2.2. Hatchability Statistics

After mating, each female laid eggs in a circle. Eggs were incubated at 25 °C and 80% RH until hatching. The hatching rate for each egg circle was calculated (n = 25) using the following formula: hatching rate (%) = (number of hatched eggs/total number of eggs) × 100%.

### 2.3. Embryo Observation

KOH solution (100 mL, 20%) was added to a beaker and heated to boiling. Silkworm eggs were briefly immersed in the boiling KOH solution for 3 s and then immediately transferred to 60 °C warm water for 3 s. Subsequently, the eggs were placed in a Petri dish containing water 25 °C. Using a plastic glue head dropper, the eggs were gently blown repeatedly until intact silkworm egg embryos were isolated. Images were captured using microscope (TL3000 Ergo, Leica, Wetzlar, Germany).

### 2.4. RNA-Seq Analysis

Considering that both AS and SS were removed from cold storage (T1) simultaneously and were capable of progressing to the body pigmentation stage (T2), we collected eggs from AS and SS at both T1 and T2 for analysis (Figure 1). All samples were sequenced using the DNBSEQ platform (BGI, https://www.bgi.com/ (accessed on 20 January 2025)), generating an average of approximately 6 Gb data per sample. The raw sequencing data have been deposited in the NCBI database (BioProject: PRJNA1212802). The eggs were collected and mixed evenly, and 200 mg of each sample was weighed for total RNAs isolation. The mRNA from each sample was extracted from more than three individuals, with three biological replicates. SOAPnuke (v2.3.0) software was used to remove low-quality and contamination data. The obtained valid data (clean reads) were aligned to the silkworm reference genome and reference gene (NCBI GenBank: GCF_014905235.1) using HISAT2 (v2.2.1) and Bowtie2 (v2.5.1) software. The DEGseq algorithm was used to detect differentially expressed genes. Significantly differentially expressed genes were those whose gene expression level was |log_2_ (fold change)| ≥ 1, and false discovery rate (FDR) correction was performed for *p*-value, FDR ≤ 0.001. The DEGs were classified by the Gene Ontology (GO) and Kyoto Encyclopedia of Genes and Genomes (KEGG).

### 2.5. RNA Isolation, cDNA Synthesis, and qPCR Analysis

Total RNAs were isolated from eggs using Trizol^®^ reagent (Invitrogen, Carlsbad, CA, USA). The RNA concentration and integrity were measured using an ultra-micro spectrophotometer (Nano-330, Allsheng, Hangzhou, China). An amount of 1 µg of total RNAs was used with the RevertAid™ First Strand cDNA Synthesis Kit (Thermo Fisher Scientific, Waltham, MA, USA) for complementary DNA (cDNA) synthesis. Quantitative real-time PCR (qRT-PCR) analyses were performed using a SYBR Green Realtime PCR Master Mix (Thermo Fisher Scientific, Waltham, MA, USA). The PCR conditions were as follows: initial incubation at 95 °C for 5 min, 35 cycles at 95 °C for 15 s, and 60 °C for 1 min. The *B. mori* gene encoding *ribosomal protein 49* (*Bmrp49*) was used as an internal control [29,30]. A relative quantitative method (^△△^Ct) was used to evaluate quantitative variation, and each sample was repeated three times. The gene-specific primers used for qRT-PCR were listed in Table 1.

### 2.6. Phylogenetic Analysis

Evolutionary relationships of PPARs protein among representative species were inferred using the Neighbor-Joining method. And evolutionary analyses were conducted in MEGA 11 [31,32]. Tests were performed by Bootstrap (1000 replicates). Evolutionary distances were computed using the Poisson correction method.

### 2.7. Determination of Fatty Acids

A total of 0.1 g of eggs were accurately weighed and thoroughly homogenized. Subsequently, 1 mL of petroleum ether (64742-49-0, Aladdin, Shanghai, China), 1 mL of diethyl ether (60-29-7, Nanjing Reagent), and 1 mL of 0.4 mol/L KOH.CH3OH were added to the homogenate, followed by thorough mixing. The mixture was then shaken vigorously, sealed, and incubated overnight at room temperature. After incubation, a saturated NaCl solution was added, and the mixture was allowed to stand for 20 min to facilitate phase separation. The supernatant was carefully collected following stratification. An additional 1 mL of petroleum ether was added, and the sample was centrifuged at 6000 r/min for 30 min to complete the extraction process. The supernatant was filtered with a 0.22 µL filter membrane. The fatty acid content was determined by gas chromatography mass spectrometry (GC-MS, 7890A, Agilent, Palo Alto, CA, USA). The gas chromatography (GC) analysis was performed using an HP-88 capillary column (30 m × 0.25 mm, 0.20 µm) with an inlet temperature set at 270 °C. The oven temperature was initially held at 70 °C for 1 min, followed by a gradual increase to 230 °C at a rate of 10 °C/min, and maintained at 230 °C for 5 min. The MS conditions were as follows: the ion source temperature was 230 °C, the ionization mode was EI, the electron energy was 70 eV, the quadrupole ion source temperature was 150 °C, and the scanning range *m*/*z* was 33~350.

### 2.8. Artificial Regulation of PPARs Activity

A well-developed and simultaneously pupated individual was selected and placed at 25 °C and 80% RH. The inhibitors of PPARs were injected into the pupa using a microsyringe, including PPARα inhibitor (HY-15372, MCE, Monmouth Junction, NJ, USA), PPARβ/δ inhibitor (HY-15577, MCE, NJ, USA), and PPARγ inhibitor (HY-N0292, MCE, NJ, USA). The injection was performed by vertically inserting the needle into the middle of the third somite on the abdomen. The pupal stage of the QiufengA strain lasts 13 days, and the injection was given on the 12th day (one day before emergence). DMSO:Oril (1:9, *v*/*v*) was used as a solvent to configure different concentrations of inhibitors. According to the characteristics of inhibitors and the preliminary experimental concentration test, three optimal concentrations were finally set for each inhibitor. The PPARα and PPARβ/δ inhibitors were tested at 80 µM, 160 µM, and 320 µM, while the PPARγ inhibitor concentrations were set at 20 µM, 40 µM, and 80 µM. Eggs were collected 24 h after inhibitor injection, and enzyme activity was detected using ELISA kit (YJ320345, YuanjuBio, Shanghai, China).

### 2.9. mRNA Synthesis and Cell Transfection

Two single-guide RNA (sgRNA) target sites were designed according to the 5′-GGNGG-3′ principle, including ‘GGCAGCCTTCTTGGTATTGTGG’ and ‘GGGGCGGCTACTGTTGAGAAGG’. The sgRNA templates were synthesized using oligonucleotides encoding the T7 polymerase binding site, which were then annealed to common oligonucleotides that encode the remainder of the sgRNA sequence. The reaction conditions were as described previously [33]. sgRNAs were synthesized in vitro using the MEGAscript Kit (AM1333, Invitrogen, Carlsbad, CA, USA). Cas9 mRNAs were synthesized using the Mmessage mMACHINE kit (Ambion). The sgRNA-specific primers used for plasmid construction were listed in Table 1.

sgRNAs and Cas9 mRNAs were independently transfected into BmN cells using the lipofectamine reagent (Invitrogen, Carlsbad, CA, USA) following the manufacturer’s protocol. For transfection, 500 μM of each RNA was transfected into BmN cells in a 6-well cell culture plate in triplicate. Following transfection, the BmN cells were cultured in TC-100 insect medium (LVN1013, Livning, Beijing, China) at 27 °C.

### 2.10. Cell Viability Assay and Oil Red Staining

After transfection for 48 h, 100 µL of cell suspension was added to a 96-well cell culture plate containing 10 µL of CCK-8 solution (40203ES60, YEASEN, Shanghai, China) in triplicate. The plate was then cultured at 27.5 °C for 2 h, after which an absorbance at 450 nm was determined to assess cell viability. The cells were collected and stained with Oil Red kit (C0158, Beyotime, Shanghai, China) to observe the lipid drops after transfection for 48 h. The culture medium was discarded and fixed with 4% paraformaldehyde for 10 min, followed by two washes with PBS. A total of 200 µL of dyeing washing solution was added and soaked for 20 s. Then, oil red O dyeing solution was added to stain for 20 min. After washing, the cell lipid droplets were investigated under microscope (BX51, Olympus, Tokyo, Japan), and the area of lipid droplets was calculated by ImageJ 1.5.4 software.

### 2.11. Statistical Analysis

Statistical analysis was performed using GraphPad Prism 8.3.0. A two-tailed Student’s *t*-test was applied to compare experimental groups. Three independent replicates were used for each treatment. Means were determined, and error bars show the means ± SEM.

## 3. Results

### 3.1. Hatching Rate and Embryo Development of QiufengA

**Figure 2:**
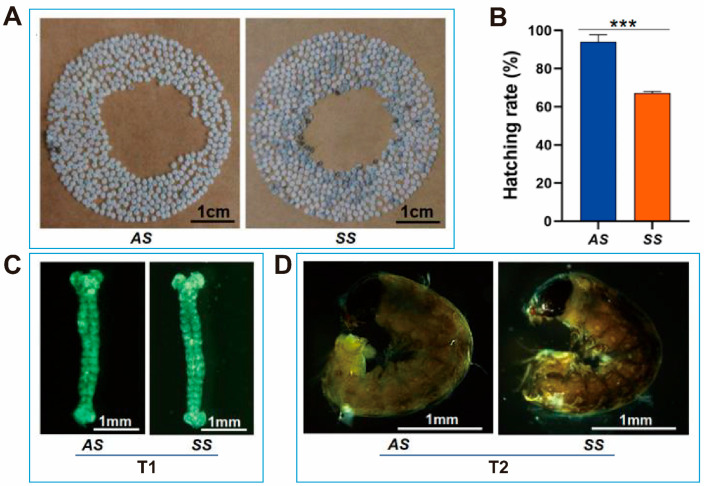
Embryonic development and morphology in silkworm production strain QiufengA. (**A**) Hatching of AS and SS. The white was the eggshell after hatching, and the blue-gray was the unhatched egg. (**B**) Hatching rate of AS and SS. (**C**) Prop-2 embryo. (**D**) Hex-5 embryo. Abbreviations: autumn-produced eggs for next spring rearing (AS), spring-produced eggs for next spring rearing (SS). The data shown are means ± S.E.M. (n = 3). Asterisks indicate significant differences with a two-tailed *t*-test: *** *p* < 0.001.

There was a significant difference in hatching rate between AS and SS in silkworm production strain QiufengA (Figure 2A). The hatching rate of AS reached 94.04%, whereas that of SS was only about 67.17%, (Figure 2B). Both AS and SS were removed from cold storage at the same time (T1) and were able to develop to the body pigmentation stage (T2). At T1, AS and SS had developed into the prop-2 embryo stage (the longest stage) (Figure 2C). During this stage, the embryo exhibited a slender body, well-developed head folds, a slightly deeper depression, 18 distinct somites, and the initial formation of a longitudinal groove between the first and second somites. Both AS and SS had developed to the hex-5 embryo (the body pigmentation stage) at T2 (Figure 2D). At this stage, the embryo was fully developed, with embryonic development nearly complete and movement gradually increasing.

### 3.2. Differentially Expressed Genes of AS and SS

**Figure 3:**
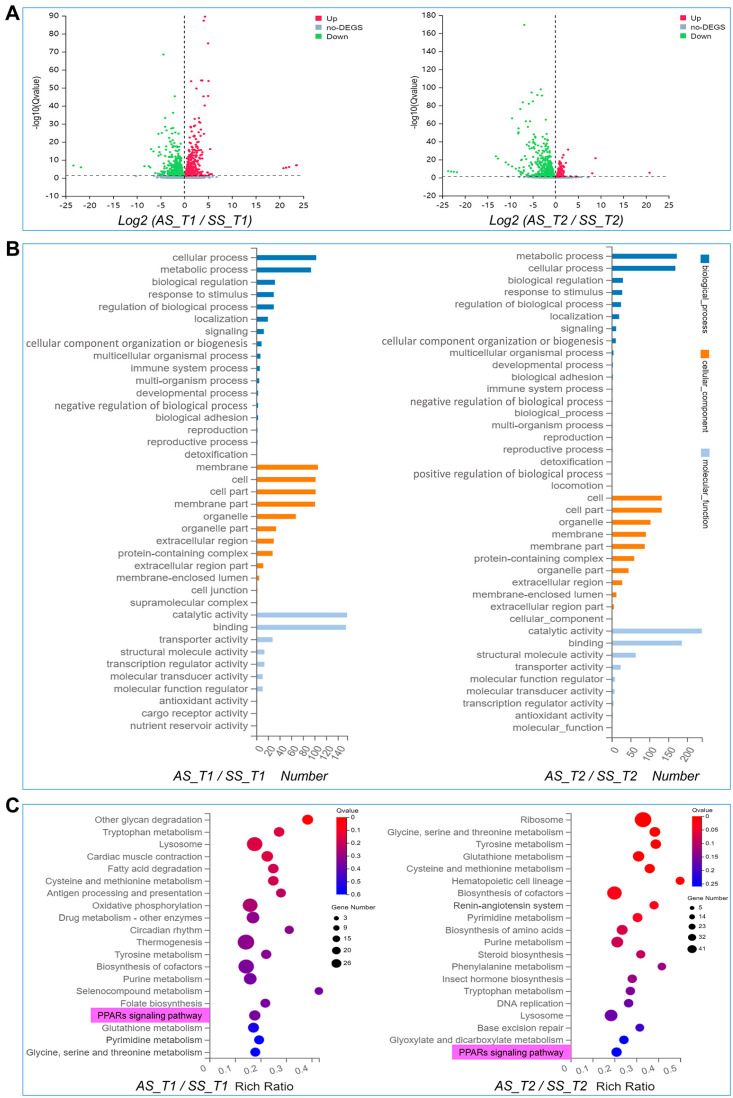
Enrichment analysis of DEGs. (**A**) DEGs volcano plot, up-regulated and down-regulated genes are shown in red and green, respectively. (**B**) GO analysis. (**C**) KEGG analysis, PPARs is shown in pink. Abbreviations: autumn-produced eggs for next spring rearing (AS), spring-produced eggs for next spring rearing (SS), Prop-2 embryo (T1), Hex-5 embryo (T2).

Based on the observed phenotypic differences, we conducted transcriptome analysis on AS and SS at both T1 and T2. There were 1209 DEGs between AS and SS at T1, including 671 up-regulated genes and 538 down-regulated genes; 1240 DEGs at T2, including 901 up-regulated genes and 339 down-regulated genes (Figure 3A). The GO classification of DEGs at T1 and T2 were primarily enriched in cell-related functions (Figure 3B). KEGG analysis showed that DEGs were mainly enriched in signal pathways related to glucose metabolism, amino acid metabolism and lipid metabolism (Figure 3C). These metabolism pathways were designed to meet the body’s energy requirements and physiological functions. Notably, DEGs were significantly enriched in the PPARs pathway at both T1 and T2, highlighting its role in cellular metabolism through energy regulation.

### 3.3. Phylogenetic Identification of PPARs Protein

**Figure 4:**
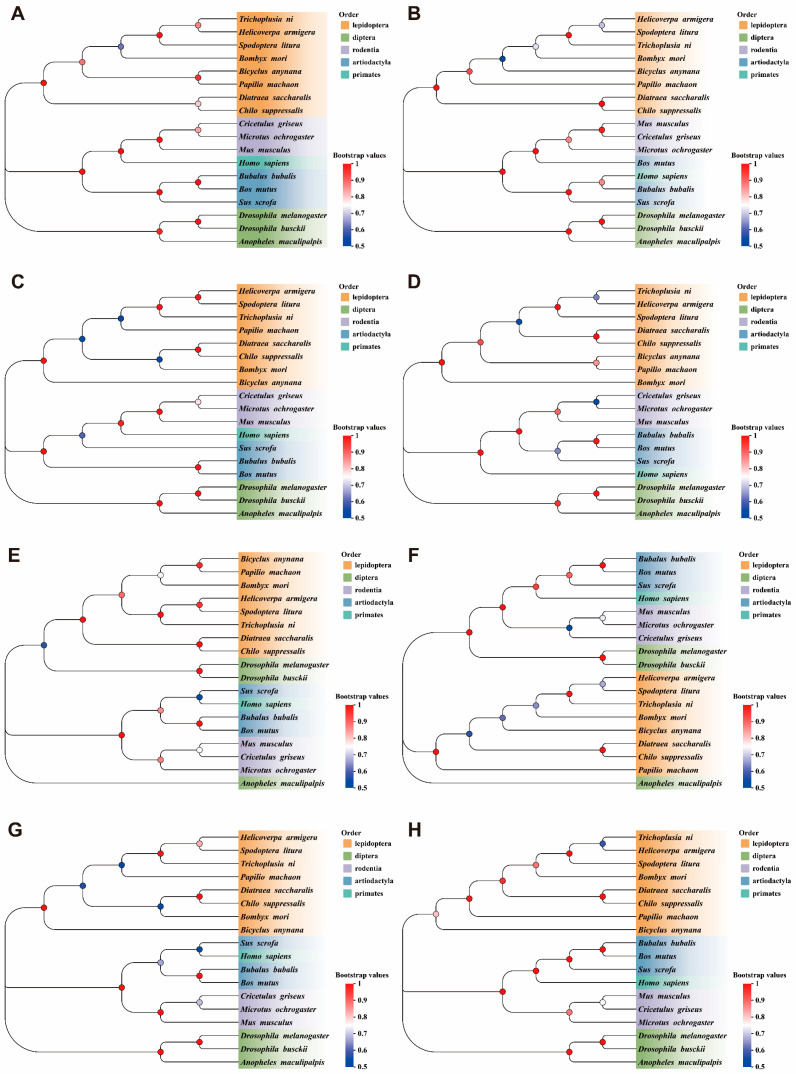
The phylogenetic relationship analysis. (**A**) BmLC-Facs5. Long-chain-fatty acid--CoA ligase 5. (**B**) BmDesat3. Acyl-CoA desaturase. (**C**) BmMe1. Malic enzyme. (**D**) BmPepck. Mitochondrial phosphoenolpyruvate carboxykinase. (**E**) BmLC-Fatp4. Long-chain fatty acid transport protein 4. (**F**) BmScd5. Stearoyl-CoA desaturase 5-like. (**G**) BmPlin. Lipid storage droplet 2-like. (**H**) BmPlin4. Perilipin-4.

We identified and analyzed the DEGs within the PPARs pathway to verify their functional consistency, including *BmLC-Facs5*, *BmDesat3*, *BmLC-Fatp4*, *BmMe1*, *BmPepck*, *BmScd5*, *BmPlin*, and *BmPlin4*. These genes were regulated by three PPAR subtypes: PPARα, PPARβ/δ, and PPARγ. The homologous sequences of the proteins encoded by these genes were selected from 18 different representative species to explore evolutionary conservation. The sequences evaluated were from Lepidoptera (*Bombyx mori*, *Trichoplusia ni*, *Bicyclus anynana*, *Helicoverpa armigera*, *Papilio machaon*, *Spodoptera litura*, *Diatraea saccharalis*, *Chilo suppressalis*), Diptera (*Drosophila melanogaster*, *Anopheles maculipalpis*, *Drosophila busckii*), Rodentia (*Mus musculus*, *Cricetulus griseus*, *Microtus ochrogaster*), Artiodactyla (*Sus scrofa*, *Bubalus bubalis*, *Bos mutus*), and Primates (*Homo sapiens*). Phylogenetic analysis indicated that these proteins are highly conserved across species (Figure 4). These data indicate that findings in silkworm regarding the function of PPARs are likely applicable to other species. The sequence numbers of the proteins in the corresponding species are listed in Table 2.

### 3.4. The Key Gene Expression Pattern of PPARs Pathway

**Figure 5:**
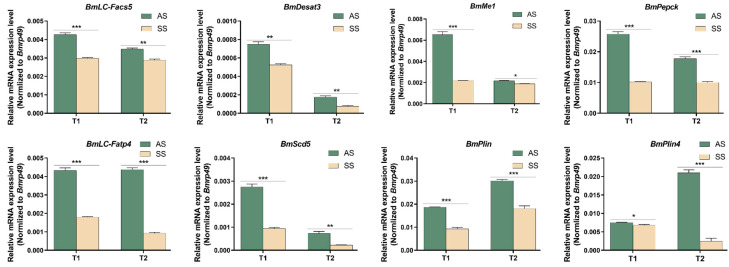
Spatial and temporal expressions of PPARs genes. Abbreviations: *BmLC-Facs5*, Long-chain-fatty acid-CoA ligase5, XM_021351606.3. *BmDesat3*, Acyl-CoA desaturase, XM_012689825.3. *BmMe1*, Malic enzyme, XM_012694093.3. *BmPepck*, Mitochondrial phosphoenolpyruvate carboxykinase, XM_038017905.2. *BmLC-Fatp4*, Long-chain fatty acid transport protein 4, XM_004929184.5. *BmScd5*, Stearoyl-CoA desaturase5-like, NM_001309595.1. *BmPlin*, Lipid storage droplet 2-like, XM_012689610.4. *BmPlin4*, Perilipin-4, XM_021348604.2. The mRNA expression level was normalized to *B. mori ribosomal protein 49* (*Bmrp49*), an internal reference. The data shown are means ± S.E.M. (n = 3). Asterisks indicate significant differences with a two-tailed *t*-test: * *p* < 0.05; ** *p* < 0.01; *** *p* < 0.001.

We detected the mRNA expression levels in AS and SS of QiufengA strain at T1 and T2. The key genes of the PPARs pathway were *BmLC-Facs5*, *BmDesat3*, *BmLC-Fatp4*, *BmMe1*, *BmPepck*, *Bmscd5*, *BmPlin*, and *BmPlin4*. At both T1 or T2, the mRNA expression of all these genes were significantly higher in AS compared to SS (Figure 5). These genes were involved in fatty metabolism and energy regulation. The storage time of SS was longer than that of AS, which was caused by the difference in fatty metabolism.

### 3.5. The Contents of Main Fatty Acids in AS and SS Were Changed

**Figure 6:**
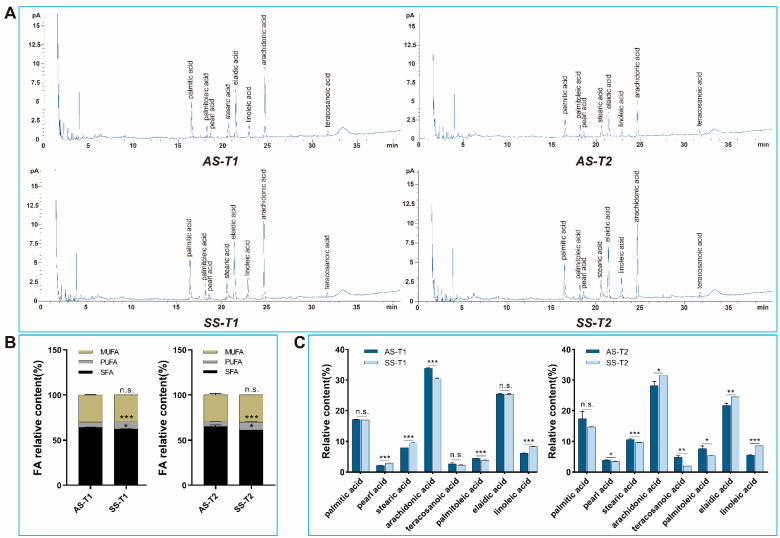
Fatty acids content in AS and SS. (**A**) GC-MS analysis. (**B**) Different types of fatty acids. (**C**) Fatty acid content changes. Abbreviations: saturated fatty acids (SFA), polyunsaturated fatty acids (PUFA), monounsaturated fatty acids (MUFA). The data shown are means ± S.E.M. (n = 3). Asterisks indicate significant differences with a two-tailed *t*-test: * *p* < 0.05; ** *p* < 0.01; *** *p* < 0.001; n.s. *p* > 0.05.

To investigate whether alterations in key lipid metabolism genes were associated with corresponding changes in the composition and abundance of major fatty acid species, we conducted a comprehensive analysis of fatty acid profiles. The main fatty acids detected were eight long-chain fatty acids in AS and SS of QiufengA strain at both T1 and T2 (Figure 6A). Among them, it contained five SFA (palmitic acid, C16:0; pearl acid, C17:0; stearic acid, C18:0; arachidonic acid, C20:0; lignoceric acid, C24:0), two MUFA (palmitoleic acid, C16:1; elaidic acid, C18:1T), and one PUFA (linoleic acid, C18:2). Compared to AS, SS exhibited a significant decrease in SFA and a significant increase in PUFA at both T1 and T2, while MUFA had no significant difference (Figure 6B). Further analysis showed that the contents of all eight fatty acids were disturbed in SS (Figure 6C). The oxidation of long-chain fatty acids was the key way for organisms to obtain energy. Different types of fatty acids played different roles in the oxidation pathway, and only a precise balance in their relative concentrations could ensure a stable supply of energy. For instance, imbalances in the levels of palmitic acid, stearic acid, and linoleic acid affected developmental capacity. The change in fatty acid content also caused the decrease in hatching rate.

### 3.6. Decreased PPARs Activity Affected Hatching of QiufengA Strain

**Figure 7:**
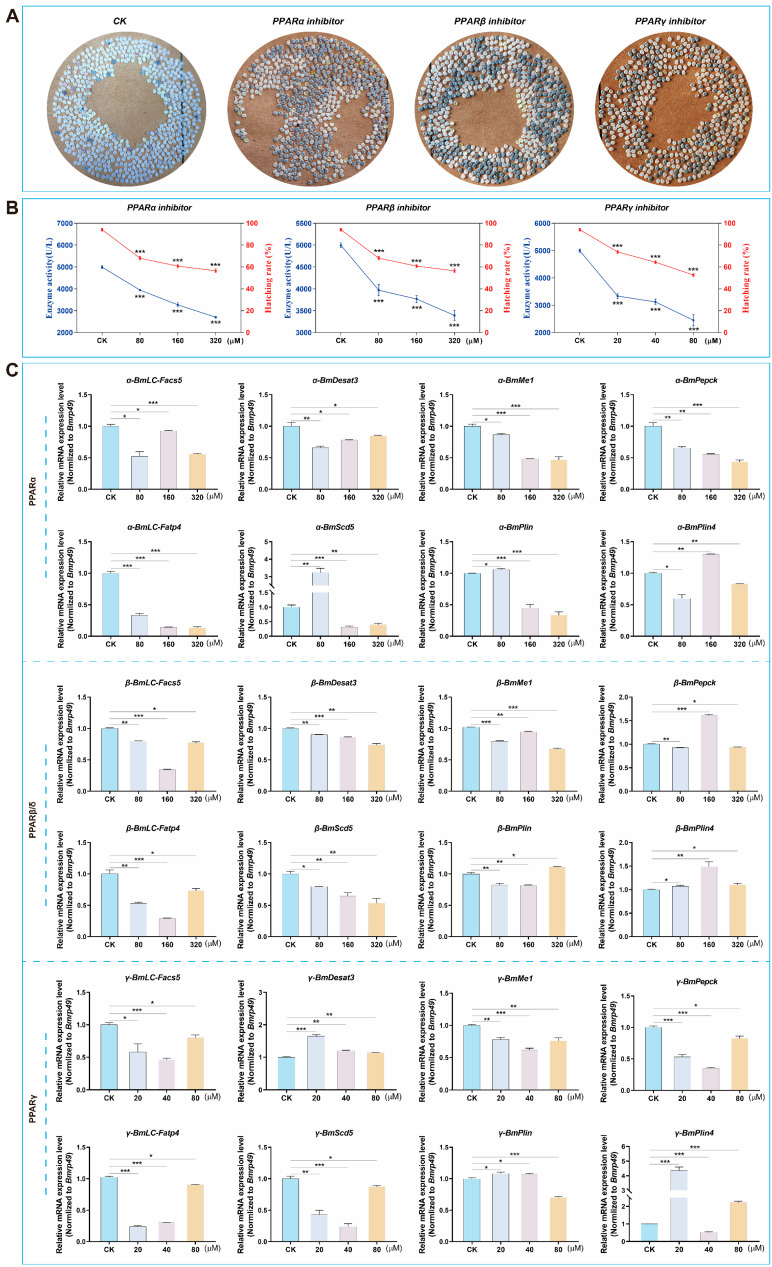
PPARs activity affected hatching. (**A**) Egg hatching. The white was the eggshell after hatching, and the blue-gray was the unhatched egg. (**B**) Enzyme activity and hatching rate. The blue was PPARs enzyme activity, and the red was hatching rate of silkworm eggs. (**C**) The mRNA expression after PPARs inhibitor treatment. Abbreviations: The CK was solvent treatment of PPAR inhibitors. *BmLC-Facs5*, Long-chain-fatty acid-CoA ligase5. *BmDesat3*, Acyl-CoA desaturase. *BmMe1*, Malic enzyme. *BmPepck*, Mitochondrial phosphoenolpyruvate carboxykinase. *BmLC-Fatp4*, Long-chain fatty acid transport protein 4. *BmScd5*, Stearoyl-CoA desaturase5-like. *BmPlin*, Lipid storage droplet 2-like. *BmPlin4*, Perilipin-4. The mRNA expression level was normalized to *B. mori ribosomal protein 49* (*Bmrp49*), an internal reference. The data shown are means ± S.E.M. (n = 3). Asterisks indicate significant differences with a two-tailed *t*-test: * *p* < 0.05; ** *p* < 0.01; *** *p* < 0.001.

The eggs laid after mating were immediately soaked with hydrochloric acid to remove diapause, and the hatching rate was observed and calculated. Compared to the control, the embryo could develop to the body pigmentation stage after inhibitor treatment, but the hatching rate was significantly reduced (Figure 7A). The hatching rate of QiufengA was 94.11% (n = 25) in the control. As the concentration of the inhibitors increased, PPARs activity decreased significantly, accompanied by a corresponding decline in hatching rates (Figure 7B). Specifically, the hatching rates after treatment with different concentrations of inhibitors were as follows: for PPARα, 68.02% (80 µM)/, 60.78% (160 µM), 56.56% (320 µM); PPARβ/δ, 73.76% (80 µM), 64.35% (160 µM), 52.71% (320 µM); PPARγ, 76.25% (20 µM), 69.25% (40 µM), 56.67% (80 µM) (n = 25). Meanwhile, we examined the mRNA expression levels of PPARs key genes after treatment with different inhibitors. We found that the expression of these genes was disrupted (Figure 7C). This may lead to lipid metabolism and energy regulation disorders, which ultimately lead to the main cause of hatching obstruction.

### 3.7. Knockdown the Key Gene BmPlin4 Affected Lipid Homeostasis in BmN Cells

**Figure 8:**
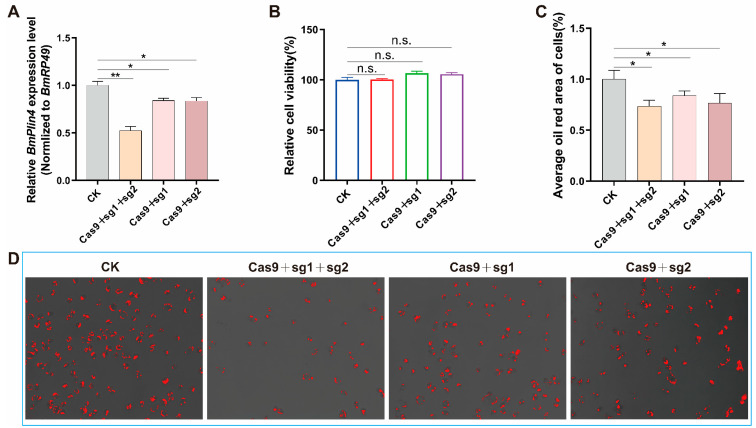
Knockdown of *BmPlin4* gene affected lipid metabolism. (**A**) The mRNA expression of *BmPlin4* gene. (**B**) Cell viability. (**C**) lipid droplet content of cells. (**D**) Oil red O staining and quantification using ImageJ 1.5.4. The data shown are means ± S.E.M. (n = 3). Asterisks indicate significant differences with a two-tailed *t*-test: * *p* < 0.05; ** *p* < 0.01; n.s. *p* > 0.05.

We selected *BmPlin4* as the representative gene due to its central role in the PPARs pathway, directly interacting with PPARα, PPARβ/δ, and PPARγ. To investigate its function, we knocked down *BmPlin4* in cells using CRISPR/Cas9. The mRNA expression of *BmPlin4* gene was significantly down-regulated after transfection with Cas9 and sgRNA, with the double-target knockdown showing the most pronounced effect (Figure 8A). In addition, down-regulation of *BmPlin4* gene did not affect cell viability (Figure 8B). Oil red staining showed that the downregulation of *BmPlin4* reduced the lipid droplet (LD) content within the cells (Figure 8C,D). The lipid droplet played an important role in the regulation of energy balance and lipid metabolism. The change in lipid droplet content directly affected embryonic development. These findings further support the hypothesis that down-regulation of key genes in the PPARs pathway disrupts cellular lipid metabolism, thereby impairing hatching.

## 4. Discussion

**Figure 9:**
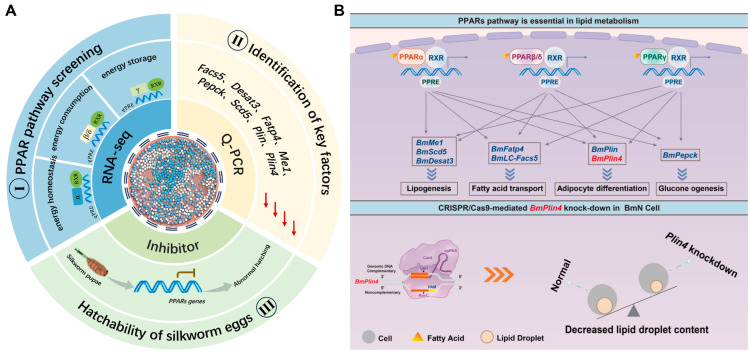
Hatching regulation of QiufengA strain. (**A**) The research content, including I, II, III part of the content. (**B**) PPARs regulated lipid metabolism patterns. PPARs affected lipid metabolism through network regulation of key genes. Knocking down the BmPlin4 gene resulted in a reduction in lipid droplets content, further demonstrating that PPARs was critical in lipid metabolism.

Our study provides genetic evidence for the hatching differences between the AS and SS of the silkworm production strain QiufengA, underscoring the crucial role of the PPARs pathway in hatching regulation (Figure 9A). Transcriptomic analysis reveals that the differentially expressed genes (DEGs) are predominantly associated with cellular metabolism and energy regulation at T1 and T2. Embryonic development, characterized by intricate processes of cell division and differentiation leading to tissue formation [10,11], fundamentally relies on efficient metabolic pathways to maintain adequate energy supply [34].

PPARs, a family of ligand-activated transcription factors, serving as master regulators of lipid metabolism, energy balance, and cell differentiation [35,36,37]. PPARs comprise three subtypes (PPARα, PPARβ/δ, and PPARγ) that regulate a network of metabolic genes, including *BmLC-Facs5*, *BmDesat3*, *BmMe1*, *BmPepck*, *BmLC-Fatp4*, *BmScd5*, *BmPlin*, and *BmPlin4* [38,39,40,41]. These genes demonstrate functional conservation across species, influencing lipid metabolism, energy homeostasis, and embryonic development. For instance, *LC-Facs5* homolog deletion in *Mus musculus* disrupts fat metabolism [42], while *Desat3* upregulation in silkworms correlates with embryonic development [43]. *Me1* manipulation affects fat storage and redox homeostasis [44,45], and *Pepck* downregulation in *Drosophila* reduces triglyceride accumulation [46,47,48]. PPARs demonstrates remarkable functional conservation across diverse species, regulating lipid metabolism during wing development in *Acyrthosiphon pisum* [38], mitochondrial fatty acid transport in *Mus musculus* [49], and overwintering survival in *Culex pipiens* [50]. Key components like *LC-Fatp4* and *Plin* proteins maintain lipid homeostasis, with deficiencies leading to developmental defects and reduced viability across species [51,52,53,54,55,56,57,58,59].

PPARs provide the necessary energy for the individual. A decrease in PPARs activity hampers females’ ability to obtain the energy needed for overwintering, ultimately leading to reduced egg production and hatching rates [60,61,62]. In QiufengA, extended SS storage duration resulted in PPARs pathway genes downregulation and energy imbalance. Lipid droplets, dynamic organelles central to energy storage and cellular metabolism [62], are significantly affected by PPAR-mediated regulation (Figure 9B). Perilipin (Plin) proteins, particularly Plin4, play crucial roles in lipid droplet dynamics. Plin4 serves as a reservoir for shell proteins, facilitating triglyceride packaging and maximizing energy storage [63,64]. While *BmPlin4* knockdown in BmN cells maintains cell viability, it significantly reduces lipid droplet content, highlighting its role in lipid droplet stability. *Plin4* knockout mice exhibited a marked decrease in adipose tissue mass. Consistent with this phenotype, *Plin4*-deficient iWAT1 cells demonstrated abnormal lipid droplet morphology and distribution, providing compelling evidence for Plin4’s crucial involvement in lipid droplet dynamics and lipid metabolism [64]. As essential energy reservoirs, lipid droplets support embryonic development through regulated lipid mobilization [65,66]. In *Drosophila* embryos, lipid droplet-derived lipids and proteins provide essential substrates for embryonic cell proliferation and differentiation [67], with metabolic reprogramming ensuring stage-specific energy supply [68,69].

PPARs play an important role in regulating the metabolism and content of saturated fatty acids (SFA) and unsaturated fatty acids (UFA). PPARs affect the proportion of SFA, PUFA, and MUFA in cells and tissues by regulating the expression of genes involved in fatty acid metabolism [70,71]. The duration of silkworm storage during diapause affects PPARs, thereby inducing moderate changes in the content of various fatty acids. While AS and SS share identical fatty acid compositions, quantitative differences significantly impact hatching rates. Key fatty acids demonstrate stage-specific variations: palmitic acid (C16:0) and stearic acid (C18:0), essential for lipid droplet formation [72], are reduced in SS at both T1 and T2 stages. Linoleic acid (C18:2), known to inhibit oocyte maturation [73], shows differential accumulation patterns. Insects adapt to thermal stress through PPAR-mediated regulation of fatty acid composition [74,75]. Low-temperature adaptation requires optimal PUFA levels to maintain membrane fluidity [76,77], while high-temperature conditions necessitate balanced SFA for membrane stability [78]. However, extreme PUFA or SFA levels disrupt cellular homeostasis, impairing embryonic development [79,80,81]. In QiufengA, SS eggs exhibit temperature-dependent fatty acid imbalances: elevated PUFA at low-temperature T1 and reduced SFA at high-temperature T2, ultimately leading to hatching failure.

Our findings establish PPARs as a central regulator of silkworm egg hatching, with decreased activity disrupting fatty acid metabolism and energy homeostasis. This research not only identifies potential targets for artificial hatching control but also enhances our understanding of embryonic development regulation. The evolutionary conservation of PPARs pathway genes suggests broad applicability of these findings, contributing to improved sericulture production efficiency and population control strategies.

## Figures and Tables

**Table 1:** Specific primers.

Primer Name	Primer Sequence (5′-3′)
*qRT-PCR*	
BmLC-Facs5-F	CGTTGTAGCTTCGATGTGCA
Bm LC-Facs5-R	CCAGCCTCCTTATATCGCCA
BmDesat3-F	CACAGACTTTGGGCACACAA
BmDesat3-R	GGCTTTGATTTCGGGATGCT
BmMe1-F	ACTGCACTTTCAACGACGAC
BmMe1-R	TTCTTGACGATGAGGCCCTT
BmPepck-F	AATCTCGCAATGATGACGCC
BmPepck-R	TTTTGAACACCGTCGCCATT
BmLC-Fatp4-F	TGTGATCAGGCCCAAGTTCT
BmLC-Fatp4-R	CCGGTCCACTATTTGTTGCC
BmScd5-F	TGAGTGACCTTCAAGCGGAT
BmScd5-R	CGCACTATTGACCAGCCAAG
BmPlin-F	TTGGACCACTATCTACCGCC
BmPlin-R	ACCCGAGTTTCTGAGTTCGT
BmPlin4-F	CGAAGACGCCAAAGAGAAGG
BmPlin4-R	TACTTGTCGGCGTCCTTGAT
BmRP49-F	TCAATCGGATCGCTATGACA
BmRP49-R	ATGACGGGTCTTCTTGTTGG
*Plasmid construction*	
sgRNA-F1	TAATACGACTCACTATAGGCAGCCTTCTTGGTATTGTGTTTTAGAGCTAGAAATAGCAA
sgRNA-F2	TAATACGACTCACTATAGGGGCGGCTACTGTTGAGAAGTTTTAGAGCTAGAAATAGCAA
sgRNA-R	AGCACCGACTCGGTGCCACTTTTTCAAGTTGATAACGGACTAGCCTTATTTTAACTTGCTATTTCTAGCT

**Table 2:** The protein sequence number.

Species Name	Sequence Number
*Bombyx mori*	XP_012550651.2, XP_012545279.1, XP_012549547.1, XP_037873833.1, XP_004929241.1, NP_001296524.1, XP_012545064.1, XP_021204279.2,
*Trichoplusia ni*	XP_026724857.1, XP_026732544.1, XP_026725998.1, XP_02672833.1, XP_02673812.1, XP_026744351, XP_026727208.1, XP_026739507.1,
*Bicyclus anynana*	XP_023938956.1, XP_023935398.1, XP_023943582.1, XP_023942610.2, XP_023953014.1, XP_052739993.1, XP_023948300.1, XP_052741276.1,
*Helicoverpa armigera*	XP_021190129.1, XP_02118361.3, XP_063893783.1, XP_063897571.1, XP_049706614.2, XP_021195974.3, XP_021195577.3, XP_063892118.1,
*Papilio machaon*	XP_014360141.1, XP_014362552.2, XP_045535389.1, XP_0455405334.1, XP_014362762.2, KPJ14531.1, XP_045535146.1, XP_045536721.1,
*Spodoptera litura*	XP_022822534.1, XP_022814607.1, XP_022825750.1, XP_022818283.1, XP_0228204601, XP_022825758.1, XP_022826143.1, XP_022834648.1,
*Diatraea saccharalis*	CAH0755737.1, CAG9785496.1, CAG9787336.1, CAG9792994.1, CAG9784543.1, CAG9787313.1, CAG9787424.1, CAH0751628.1,
*Chilo suppressalis*	RVE52369.1, CAH0407053.1, CAH0399100.1, RVE51794.1, RVE51223.1, RVE48477.1, CAH2981644.1, CAH0398210.1,
*Drosophila melanogaster*	NP_649067.2, NP_652731.1, NP_5248802, NP_611341.1, Np_001260354.1, CAB69054.1, NP_001036276.1, NP_001286253.1,
*Anopheles maculipalpis*	XP_050080276.1, XP_050070393.1, XP_05006733.1, XP_050069062.1, XP_050068915.1, XP_050069472.1, XP_050071398.1, XP_050067548.1,
*Drosophila busckii*	XP_017842548.1, ALC47890.1, ALC46605.1, XP_017838813.1, XP_017835074.2, XP_017846257.1, XP_017853279.1, ALC40480.1,
*Mus musculus*	NP_082252.1, AAA40103.1, NP_852072.2, EDL36290.1, NP_03611.1, 4YMK_A, BAC27409.1, NP_001397092.1,
*Cricetulus griseus*	XP_007650740.1, XP_027263037.1, XP_027263401.1, XP_003502116.1, XP_035292398.1, XP_003508493.1, XP_035297263.1, XP_027272679.1,
*Microtus ochrogaster*	XP_005352541.1, KAH0504555.1, KAH0500186.1, KAH0509637.1, XP_005346222.1, XP_005352393.1, KAH0505204.1, XP_005357949.2,
*Sus scrofa*	XP_005671765.1, NP_001107750.1, NP_001231187.1, NP_001155225.1, XP_013849357.2, NP_998946.1, AEZ36149.1, XP_020939716.1,
*Bubalus bubalis*	AIU41596.1, XP_025145597.1, XP_025141974.3, XP_025151907.1, Xp_006065433.2, NP_001277844.1, XP_044789244.1, XP_006067173.3,
*Bos mutus*	XP_005906616.1, XP_005892117.1, MXQ91848.1, XP_005904960.1, XP_005908530.1, XP_005892117.1, MXQ82814.1, MXQ83873.1),
*Homo sapiens*	KAI4077466.1, NP_001032671.2, AAC50613.1, NP_004554.3, NP_005085.2, CAA73998.1, KAI2575794.1, NP_001354797.1,

## Data Availability

The data that support the findings of this study are available on request from the corresponding author.
